# Proliferative capacity and cytokine production by cells of HIV-infected and uninfected adults with different helminth infection phenotypes in South Africa

**DOI:** 10.1186/1471-2334-14-499

**Published:** 2014-09-11

**Authors:** Zilungile L Mkhize-Kwitshana, Musawenkosi LH Mabaso, Gerhard Walzl

**Affiliations:** School of Laboratory Medicine and Medical Sciences, College of Health Sciences, University of KwaZulu-Natal, P.O. Box 7, Congella, 4001 South Africa; HIV/AIDS, STI and TB, Human Sciences Research Council, Private Bag X07, Dalbridge, Durban, 4014 South Africa; Division of Molecular Biology and Human Genetics, MRC Centre for Molecular and Cellular Biology, DST and NRF Centre of Excellence for Biomedical TB Research, Faculty of Medicine and Health Sciences, Stellenbosch University, Cape Town, South Africa

**Keywords:** HIV, Helminths, Co-infection, Proliferation, Ki67, CTLA-4, Cytokines

## Abstract

**Background:**

It has been suggested that the proliferative capacity of cells from individuals with HIV or both HIV and helminth infections is attenuated and cytokine production is dysregulated. This study describes peripheral blood mononuclear cell proliferation capacity and cytokine profile from individuals with HIV or both HIV and helminth infections in South Africa.

**Methods:**

Forty HIV-infected and 22 HIV-uninfected participants were randomly selected and stratified into different helminth infection phenotypes by egg excretion and *Ascaris lumbricoides* specific –immunoglobulin-E (IgE) levels. Five day cell cultures of participants, unstimulated or stimulated with Phytohaemaglutinnin, Streptokinase, HIV-1 p24 and *Ascaris lumbricoides* worm antigens were stained with monoclonal antibody-fluorochrome conjugates (Ki67-FITC and CTLA-APC-4). Percentage expression of Ki67 and CTLA-4 was measured to determine cell proliferation and regulation, respectively. Culture supernatants were analysed for the expression of 13 cytokines using the Bioplex (BioRad) system. Kruskal Wallis was used to test for differences in variables between helminth infected subgroups who were either having eggs in stool and high IgE (egg^+^IgE^hi^); or eggs in stool and low IgE (egg^+^IgE^lo^); or no eggs in stool and high IgE (egg^-^IgE^hi^) and those without helminth infection (egg^-^IgE^lo^).

**Results:**

Individuals excreting eggs in stool with high serum IgE (egg^+^IgE^hi^ phenotype) had potent mitogen responses but consistently produced low, but statistically non-significant antigen–specific (HIV^-^1 p24 (p = 0.41) and *Ascaris* (p = 0.19) and recall antigen (Streptokinase; p = 0.31) Ki67 responses. The group also had reduced type 1 cytokines. Individuals excreting eggs in stool with low serum IgE( egg^+^IgE^lo^ phenotype) had a more favourable antiviral profile, characterized by higher IFNγ, IL-2, lower IL-4 and higher IL-10 production.

**Conclusion:**

The findings suggest that dual HIV/helminth infection with egg excretion and/or high *Ascaris* IgE phenotye may be linked with poor proliferative capacity and deleterious cytokine profile with regards to HIV control.

**Electronic supplementary material:**

The online version of this article (doi:10.1186/1471-2334-14-499) contains supplementary material, which is available to authorized users.

## Background

Helminths are among the most common parasitic infections affecting populations in developing countries, and have been associated with faster progression of HIV disease especially in sub-Saharan Africa [1, 2] Predominance of type 2- and/or reduced type 1-, increased pro-inflammatory cytokines, altered distribution of immune cells, increased HIV receptor molecules (CCR5, CXC-R4 and CD4), eosinophilia, induction of persistent immune activation and anergy are some of the immune modulatory factors by which helminths are suggested to promote HIV infection and disease progression [1, 3]. Both HIV and helminth infection are characterized by chronic immune activation and dysregulation of immune responses to pathogens typified by impaired capacity of immune cells to proliferate in response to antigenic stimulus [1, 4]. Furthermore, it has been proposed that the predominance of type 2 cytokines induced by helmiths downregulates type 1 responses [1, 5] which are critical for the control of HIV infection. In addition, although still inconclusive to date, HIV disease progression has long been associated with increased type 2 or reduction of type 1 cytokines [6]. It is therefore expected that dual infection would have an additive effect on the immune defects associated with the individual infections. It is noted, however that despite attempts to derive scientifically conclusive evidence of a positive impact of deworming on HIV progression, studies have, in the main, reported conflicting results [7]. The current study focuses on immunological impact of co-infection, without looking at the effect of deworming. The aim is to further define immunological effects associated with helminth infection in the context of HIV progression parameters in order to extend this body of knowledge.

The human Ki-67 protein, up-regulated during active cell cycle phases, has been shown to be a reliable and specific quantitative marker of cell proliferation [8] or indicator of cell turnover during chronic infections such as HIV [9]. The protein is expressed by proliferating cells at G1, S and M phases of the cell cycle [10]. On the other hand, the down-regulatory molecule, cytotoxic T lymphocyte associated antigen 4 (CTLA-4) inhibits cell cycle progression through reduction of cyclin-dependant kinases [11] to maintain T cell homeostasis.

In South Africa (SA), a large proportion of the population is exposed to both HIV and helminth infections particularly among those living under conditions of poverty [12]. However, there is limited research undertaken to evaluate the immunological effects of dual infection in these settings. It is important to investigate the deleterious effects of co-infection with HIV and helminths in the country, where the latter are largely neglected, and HIV is at pandemic proportions. Such research may strengthen advocacy for regular mass deworming in line with the World Health Assembly 54.19 recommendation of 2001 [13], and as part of a holistic approach to HIV control and prevention [2, 13].

Previously we reported that different helminth infection phenotypes display heterogeneity of immune responses during HIV and helminth co-infection. Irregular distribution of immune cells and highly activated immune profiles were described in a specific group of individuals with dual infection [14]. The present study further describes peripheral blood mononuclear cell proliferation capacity as measured by Ki67 expression, as well as cytokine profiles in the same HIV-infected and negative South African adults with different helminth infection phenotypes in order to determine if cells from dually infected individuals have skewed cytokine production and more impaired capacity to proliferate in response to antigen stimulation.

## Methods

### Study participants

The current analysis is part of a bigger study that was approved by the SA Medical Research Council and Stellenbosch University Ethics Committees (N04/02/045) in 2002 and 2004 respectively. Written informed consent was obtained from all participants. The recruitment of participants has been described in detail elsewhere [12, 14]. Essentially participants were recruited from a low resource informal settlement of Khayelitsha in the Western Cape province of SA from May 2002 to November 2003 as part of a cohort study to assess the effects of regular deworming of HIV-helminth co-infected individuals. The selected HIV-infected patients were attending an HIV-support group at the Mathew Goniwe Clinic, where they received clinical care and the HIV-uninfected were recruited from individuals accompanying patients to the Clinic. Due to budgetary constraints for the current analyses, of the 124 HIV^+^ eligible participants previously described [14], forty participants (ten in each of the four subgroups described below) were systematically randomized for selection and all the twenty-two HIV-uninfected, eligible individuals were included by default. At the time of study recruitment, antiretroviral treatment (ART) was not available at public health institutions in South Africa. Hence, the HIV-1 positive participants were antiretroviaral therapy-naïve. In addition, the unavailability of ART made it difficult to recruit HIV-uninfected individuals. Participants with other infectious and systemic immune suppressive diseases, those on Bactrim prophylaxis and vitamin supplementations, had been pregnant in the previous six months before study commencement, were using illicit drugs or excessive alcohol were excluded. Only participants eighteen years and above, infested with *Ascaris lumbricoides* and/or *Trichuris trichiura* were included.

### Helminth infection detection and classification of phenotypes

Stool samples, collected on two consecutive days were screened for intestinal parasites eggs by two independent microscopists using the formol-ether concentration [15] and the Kato Katz [16] methods respectively. If eggs were detected with one method and not detectable in the other, repeat samples were requested to confirm such discordant results. Participants were telephoned to return to the clinic and asked to submit two other sets of stools. Only individuals infected with *Ascaris lumbricoides* and *Trichuris trichiuria* were included in the study (and those infected with other helminth and protozoan parasites excluded from all analyses). Clotted blood was donated for serum *Ascaris -*specific IgE. The HIV-infected and negative groups were stratified into helminth infection subgroups according to the presence or absence of *A. lumbricoides* and/or *T. trichiura* eggs in stool and low or high *Ascaris* specific IgE. The four distinct subgroups identified comprised of three helminth –infected phenotypes: *Trichuris* and/or *Ascaris* egg positive stool and elevated *Ascaris* IgE (egg^+^IgE^hi^), a typical helminth infection; *Trichuris* and/or *Ascaris* egg positive stool without elevated *Ascaris* IgE (egg^+^IgE^lo^), modified T helper 2 response phenotype; *Trichuris* and/or *Ascaris* egg negative stool but elevated *Ascaris* IgE (egg^-^IgE^hi^) associated with a balanced Th1/Th2 Th3 response that is associated with resistance to helminth infection [17]; and those without evidence of helminth infection, *Trichuris* and *Ascaris* egg negative stool with low *Ascaris* IgE (egg^-^IgE^lo^).

All individuals found to be infested by parasites were given appropriate deworming treatment (if different from the mebendazole that was given to all participants as part of the main study).

### Stimulation of peripheral blood mononuclear cells (PBMCs) cultures

Whole blood collected in cell preparation tubes with Sodium Heparin was separated by standard gradient centrifugation and cryopreserved in 10% v/v dimethylsulfoxide in RPMI 1640 (Sigma) until the experiments were done. PBMCs were retrieved by standard methods and suspended at 1×10^6^ cells/ml in RPMI (Sigma) supplemented with 2 mM glutamax (Sigma), anti-CD28 and antiCD49d (Becton-Dickenson (BD), 1 μg/ml each; 10% human male AB inactivated serum (Gibco); 2-mecarptoethanol (βME) 50 μM; penicillin 1000 U/ml and Streptomycin 100 μg/ml (Gibco). Viability was assessed by phase contrast microscopy and only cells with >90% viability used for these experiments. Total volumes of 1 ml cultures (in U bottom, 48-well polypropylene tissue culture plates, Costar) were set up in duplicates with: medium only (unstimulated); Phytohaemagluttinin (PHA) (Sigma Aldrich) 5 μg/ml; Streptokinase (Aventis) 2 μg/ml; HIV-1 p-24 ( R & D Inc.,USA) 5 μg/ml; *A. lumbricoides* crude worm antigen 10 μg/ml. The cultures were incubated at 37°C, in a humidified 5% CO_2_ incubator for 5 days. The *Ascaris-lumbricoides* crude worm antigen was prepared from female whole worms that were washed, homogenized, centrifuged for 60 mins at 4°C and serially filtered through 8 μm, 0.4 μm and 0.2 μm filters. These were aliquoted and stored at minus 80°C until use.

### Anti-Ki67 and anti-Cytotoxic T lymphocyte antigen 4 (CTLA-4) staining and flow cytometry

The capacity of PBMCs to proliferate in response to mitogen, cognate and recall antigens was determined by percentage expression of the nucleoproliferation antigen- Ki67. To differentiate between proliferating and activated cells arrested at the G_1_ cycle phase [18], the down regulatory (CTLA-4) was simultaneously determined. After culture the cells were re-suspended at 1×10^6^ cells/ml. The cells were stained with 20 μL anti-CTLA-4 monoclonal antibody (clone BN13) conjugated to Allophycocyanin (APC) (BD Pharmingen™) and subsequently fixed and permeabilised in 80% cold ethanol added dropwise, followed by intracellular staining with 20 μL of anti-Ki67 (clone B56) monoclonal antibodies directly conjugated to Fluorescein Isothiocyanate (FITC) (BD Pharmingen™) using the BD Biosciences Pharmingen™ Catalog staining protocol 556003 Rev.8 [19]. The method was modified by staining in 96 well microtiter plates and incubating the stained cells on ice in the dark for 45 minutes. The following isotype controls were included: IgG_1_k FITC and IgG_2_a APC. The samples were then analysed on a FACS Calibur flow cytometer (BD). A minimum of 10 000 events were acquired manually for each sample tube in list mode using the Cellquest software and gating in the lymphocyte region. For each individual, the unstimulated cells were used to set the gated region for all subsequent antigen- specific cells. The samples were analysed using sample ID numbers that were unlinked to the groups (subgroups) therefore the researcher was blinded during acquisition and analysis.

### Cytokine production assays in PBMCs of HIV-infected subgroups

Cytokine analyses were undertaken on HIV-infected subgroups only. Cell culture supernatants that had been harvested after five days’ culture were retrieved from -70°C freezers and allowed to cool to room temperature before assays were set up. The Human cytokine LINCOplex KIT (13-plex) assay was used as per the manufacturer’s instructions to detect a range of thirteen cytokines. Briefly, plates were BSA- blocked and washed. Cytokine standards, controls and unknown samples were added (50 μL total volumes) to 96 well plates in duplicates and incubated with antibody-immobilised micro beads. After washing, biotinylated detection antibodies were incubated with the bound cytokines. Fluorescent (Phycoerythrin- labeled) streptavidin was added. A final wash was followed by resuspension in sheath fluid for analysis in the Bioplex array reader (Bio-Rad) using the Bioplex Manager 4.1 software. Fifty beads per region were collected. A seven-point standard curve (0, 3.2, 16, 80, 400, 2000 and 10 000 pg/ml) was constructed using a 5 parameter logistic (5PL) regression and the concentration of each cytokine calculated against this curve. Unstimulated cell assays presumably approximated the relative ex-vivo baseline cytokine profiles therefore a separate analysis (of unstimulated cytokine results) was made before calculating the net antigen responses. For the antigen-specific responses, the baseline (unstimulated) values were subtracted to give the final concentration in mitogen, PHA, HIV-p24- and *Ascaris* worm-challenged cells.

### Statistical analysis

Statistical analysis was conducted in STATA version 10.0 (Stata Corporation, College Station, Texas, USA). Kruskal Wallis was used to test for differences in the medians and for multiple comparisons of all measured variables between the subgroups in HIV-infected and HIV-uninfected groups. A p-value ≤ 0.05 was considered to be statistically significant.

## Results

### Immune and virologic profile of HIV-infected and HIV-uninfected subgroups

Table 1 presents descriptive statistics of study participants. The study participants were all females. The HIV-infected were generally younger than the HIV-uninfected (median age = 28 and 41 years, respectively). The immune profiles of the HIV-infected and HIV-uninfected are described in Table 2. Compared to the HIV-uninfected subgroups (even though the numbers were much smaller in the latter), the median percentage expression of HLA-DR in CD3+ cells were more than double in the HIV-infected subgroups, while CD38 expression in CD8+ cells was more than 80% in the latter compared to that in HIV-uninfected subgroups which ranged between 27% -55%. Median CD4+ cell counts were lower among the HIV-infected groups. It is noted that among the HIV-uninfected, the HIV^-^egg^+^IgE^hi^ subgroup had the lowest median CD4+ cells though still just above the threshold for onset of immunodeficiency (0.5 cells/ml), however this was not statistically significant (p = 0.26). Among the HIV-infected subgroups, while median CD8 + CD38+ values were comparable within all the subgroups, and CD3+ HLA-DR + similar in three of the four subgroups, the HIV^+^egg^+^IgE^hi^ subgroup had the highest levels of both activation markers, with almost all (97%) CD8+ cells expressing CD38, while the median viral load in this subgroup was more than 100 000 copies/ml. The CD4+ cell levels in this subgroup were similar to the subgroup without evidence of helminth infection (egg^-^IgE^lo^) and in both cases were above 0.2 cells/ml and lower than the other two subgroups.

**Table 1:** **Demographic characteristics of study participants**

Variables	Phenotype subgroups			
	Egg^+^IgE^hi^(n = 10)	Egg^+^IgE^lo^(n = 10)	Egg^-^IgE^hi^(n = 10)	Egg^-^IgE^lo^(n = 10)
HIV-infected	Median (minimum-maximum)			
Age in years	28.2 (19.5–39.6)	34.1 (24.4–49.8)	27.15 (16.4–42.5)	27.7 (19.6–45.5)
Viral load (copies/ml)	101006.5 (7563–1013265)	77290 (1818–307768)	42274.5 (170–222371)	4234 (509–262962)
*Ascaris* epg	51.5 (0–3786)	479 (2–6799)	NA	NA
*Trichuris* epg	82 (0–265)	6.5 (0–234)	NA	NA
*Ascaris* IgE	2.375 (0.64–12.1)	0.175 (0.175–0.175)	1.23 (0.65–67.9)	0.175 (0.175–0.175)
Total IgE	823.5 (4.8–4421)	16.3 (5.2–199)	593 (76.8–1995)	67.1 (3.3–447)
**HIV-uninfected**	**Egg** ^**+**^ **Ig** ^**Ehi**^ **(n = 5)**	**Egg** ^**+**^ **Ig** ^**Elo**^ **(n = 7)**	**Egg** ^**-**^ **IgE** ^**hi**^ **(n = 3)**	**Egg** ^**-**^ **IgE** ^**lo**^ **(n = 7)**
Age in years	43.7 (28.3–74.5)	47.3 (17.9–56.2)	34.3 (20.2–40)	30.2 (22–54.2)
*Ascaris* epg	0 (0–2268)	10 (0–287)	NA	NA
*Trichuris* epg	104 (0–600)	0 (0–370)	NA	NA
*Ascaris* IgE	0.47 (0.36–2.63)	0.175 (0.18–0.3)	1.11 (0.61–4.6)	0.175 (0.18–0.3)
Total IgE	592 (106–1149)	15 (24.7–330)	674 (195–2001)	112 (59.7–874)

NA-the subgroups do not have eggs in stool; Epg-Egg per gram of stool; Total IgE- total serum Immunoglobulin-E; *Ascaris* IgE- *Ascaris lumbricoides*-specific IgE.

**Table 2:** **Immune parameters for HIV-infected and negative subgroups**

Variables	Phenotype subgroups			
	Egg^+^IgE^hi^(n = 10)	Egg^+^IgE^lo^(n = 10)	Egg^-^IgE^hi^(n = 10)	Egg^-^IgE^lo^(n = 10)
HIV-infected	Median (minimum-maximum)			
CD3 HLA-DR (%)	62 (45–84)	49 (24–84)	31 (1–66)	41 (15–57)
CD8CD38 (%)	97 (92–100)	92 (81–100)	86 (66–98)	89 (82–98)
CD4 cells/ml	0.268 (0.028–0.645)	0.4 (0.05–0.9)	0.44 (0.16–0.99)	0.222 (0.12–0.3)
Viral load (copies/ml)	101007 (7563–1 013 265)	77 290 (1818–307 768)	42 275 (170–222 371)	4234 (509–262 962)
**HIV-uninfected**	**Egg** ^**+**^ **Ig** ^**Ehi**^ **(n = 5)**	**Egg** ^**+**^ **Ig** ^**Elo**^ **(n = 7)**	**Egg** ^**-**^ **IgE** ^**hi**^ **(n = 3)**	**Egg** ^**-**^ **IgE** ^**lo**^ **(n = 7)**
CD3 HLA-DR (%)	14 (7–30)	21 (9–22)	9 (8–12)	12 (9–21)
CD8CD38 (%)	50 (41–92)	27 (18–67)	55 (38–57)	51 (17–85)
CD4 cells/ml	0.55 (0.13–0.69)	0.75 (0.43–1.2)	0.8 (0.6–1.2)	0.74 (0.4–0.94)

Egg–Faecal helminth eggs, IgE–*Ascaris lumbricoides*-specific IgE, IgE hi/lo–high/low IgE in serum.

### Ki67 expression in HIV-infected and HIV-uninfected groups

The results for the unstimulated cultures were first analysed separately to determine baseline percentage of Ki67 expression. The overall median baseline Ki67 expression was significantly higher (p = 0.01) in cell cultures of the HIV-infected group compared to the HIV-uninfected group. For the net antigen-specific results, the baseline values were subtracted from those obtained in each antigen-specific response. The net antigen specific responses were generally weaker in the HIV-infected group compared to the HIV-uninfected groups.

### Proliferation profile in subgroups of HIV-infected individuals

**Figure 1:**
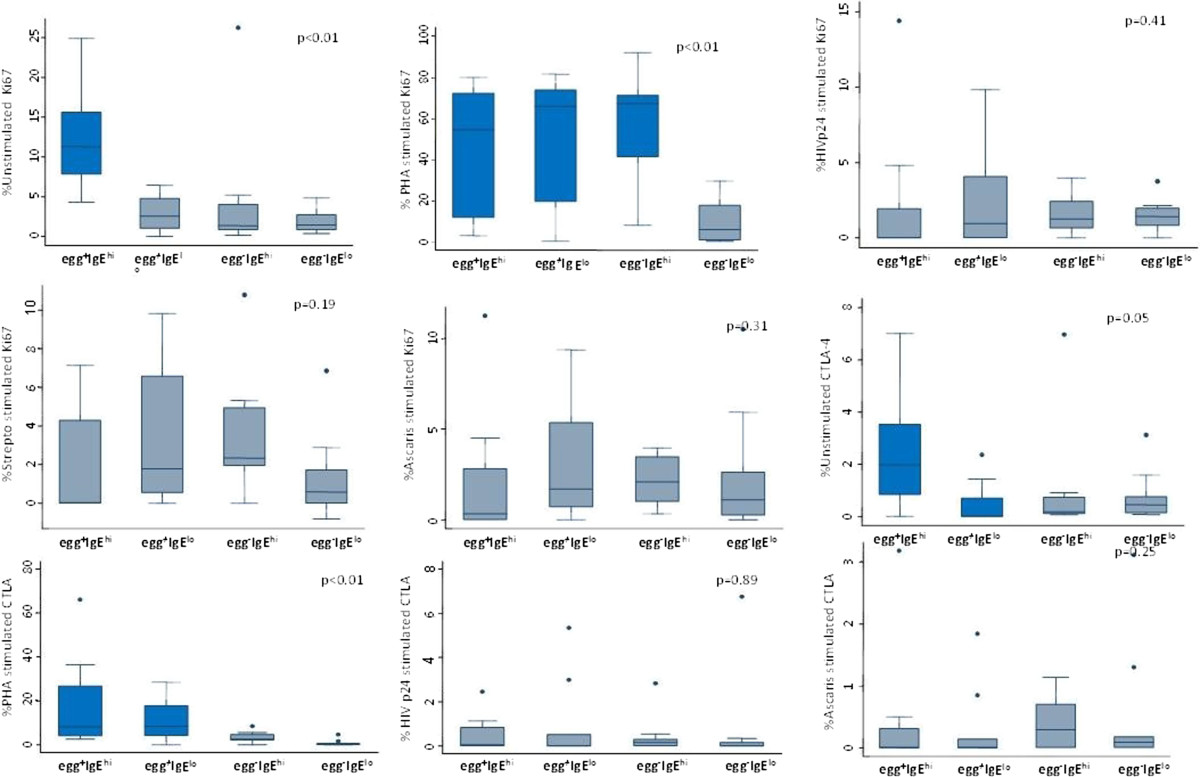
**Kruskal Wallis Box and whisker plots for proliferation antigen Ki67 and CTLA-4 antigen expression on unstimulated and antigen-stimulated lymphocytes of HIV-infected subgroups.** Significant differences between subgroups in each panel were set at p value ≤ 0.05. PHA stands for Phytohaemagluttinin-, p24 for HIV-1 p-24 antigen-, Asc for Ascaris worm- and Str for Streptokinase- antigen-stimulated cells, Egg+IgEhi denotes helminth egg positive and elevated Ascaris lumbricoides IgE subgroup, Egg+IgElo represents egg positive and low A. lumbricoides IgE subgroup, Egg-IgEhi designates egg negative and high A. lumbricoides IgE subgroup, and Egg-Iglo stands for helminth egg negative and low A. lumbricoides IgE subgroup.

Among the HIV-infected individuals, the subgroup with the egg^+^IgE^hi^ phenotype, expression of Ki67 in unstimulated cells was significantly higher (p = 0.01) than in the egg^-^IgE^lo^ and the other two subgroups. The median Ki67 expression by unstimulated cells was up to four to eight times higher in this subgroup compared to the egg^-^IgE^lo^ (p <0.01), the egg^+^IgE^lo^ (p = 0.01), and the egg^-^IgE^hi^ (p = 0.01) subgroups (Figure 1). In most instances, the expression of Ki67 was higher in unstimulated than in antigen-stimulated cells in the egg^+^IgE^hi^ subgroup such that the net antigen specific responses had a negative value or equaled zero.

The egg^+^IgE^hi^ subgroup showed a tendency towards impaired proliferative responses to recall and specific antigens but potent mitogen responses. In the HIV-infected - helminth co-infected egg^+^IgE^hi^ subgroup, while mitogen responses were high there were consistently low antigen–specific (HIV^-^1 p24 and *Ascaris*) and recall antigen (Streptokinase) Ki67 responses relative to all the other subgroups. These differences were however not significant (p = 0.41, p = 0.19 and p = 0.31) for HIV-p24, streptokinase and *Ascaris* antigens respectively) (Figure 1).

There were highly significantly stronger Ki67 responses to PHA mitogen (p = 0.01) in all subgroups with some evidence of helminth exposure (in the egg^+^IgE^hi^, egg^+^IgE^lo^ and egg^-^IgE^hi^ subgroups) compared to the egg^-^IgE^lo^ subgroup (Figure 1). The down regulatory protein, CTLA-4, was also significantly higher (p = 0.05) in unstimulated cells in the HIV-infected, egg^+^IgE^hi^ in relation to the egg^+^IgE^lo^ subgroup, and lower but not significantly, than the other two subgroups. CTLA-4 expression in response to PHA challenge was also statistically significantly higher (p = 0.01) in both groups with stool eggs (the egg^+^IgE^hi^ and egg^+^IgE^lo^) among HIV-infected groups (Figure 1).

### Proliferation profile in subgroups of HIV-uninfected individuals

**Figure 2:**
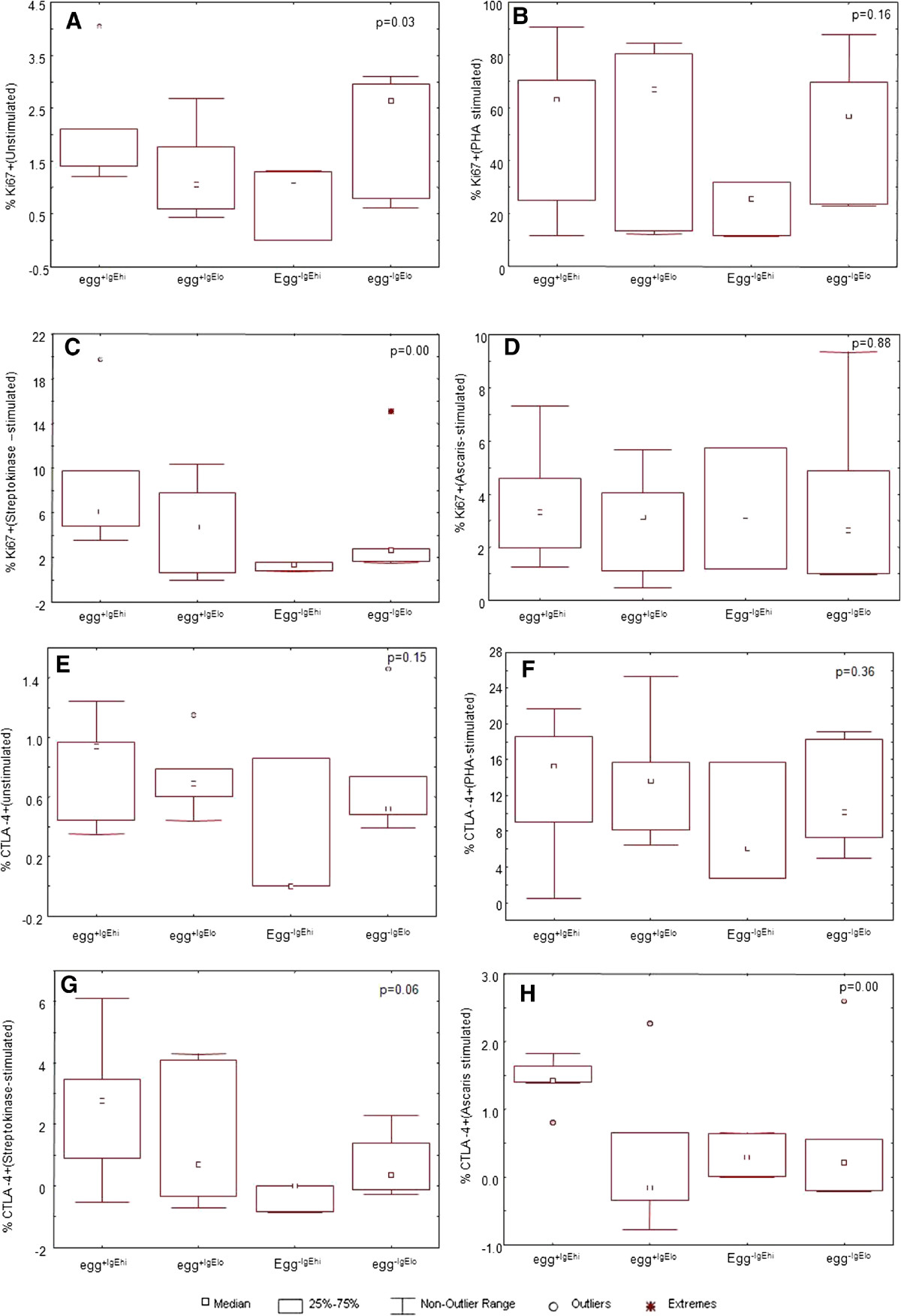
**Kruskal Wallis Box and whisker plots for proliferation antigen rowsep="1" Ki67 and CTLA-4 antigen expression on unstimulated and antigen-stimulated lymphocytes of HIV-uninfected subgroups.** Significant differences between subgroups in each panel were set at p value ≤ 0.05. PHA stands for Phytohaemagluttinin-, Asc for Ascaris worm- and Str for Streptokinase- antigen-stimulated cells, Egg^+IgEhi^ denotes helminth egg positive and elevated *Ascaris lumbricoides* IgE subgroup, Egg^+IgElo^ represents egg positive and low *A. lumbricoides* IgE subgroup, Egg^-IgEhi^ designates egg negative and high *A. lumbricoides* IgE subgroup, and Egg^-Iglo^ stands for helminth egg negative and low *A. lumbricoides* IgE subgroup.

In all HIV-uninfected subgroups almost all baseline (unstimulated) values were lower than antigen- stimulated ones. However, contrary to the responses observed in the HIV-infected participants, the baseline Ki67 expression was significantly higher (p = 0.03) in unstimulated PBMCs from the helminth-negative (egg^-^IgE^lo^) subgroup (Figure 2 Panel A).

Among the HIV-uninfected groups mitogen and antigen specific proliferative responses were not significantly different in the egg^+^IgE^hi^ when compared to the other three subgroups (Panels B, D and E). However, both egg positive subgroups (the egg^+^IgE^hi^ and the egg^+^ IgE^lo^) had significantly higher Ki67 proliferation responses to the recall antigen Streptokinase (p <0.01 Panel C) and a trend for increased CTLA-4 (p = 0.06) observed for both groups Panel G. Among HIV-uninfected subgroups CTLA-4 responses were significantly high for *Ascaris* stimulated cells in the egg^+^IgE^hi^ subgroup (p = 0.01) (Figure 2 Panel H), while these responses were similar in PHA-stimulated cells (Panel F). It was also observed that in both HIV-infected and negative groups, some Ki67 responses to *Ascaris* antigen challenge were made by the groups without faecal eggs and low specific IgE (egg^-^IgE^lo^) (medians 1.1% and 2.64% in HIV^-^ and HIV^+^, respectively).

### Cytokine responses in PBMCs

A wide range of individual levels of cytokines were obtained, therefore predominant patterns will be analysed in the following sections. In many instances the net antigen-specific responses were lower.

### Pro-inflammatory cytokines

The pro-inflammatory cytokines IL-6 and IL-8 were very high in unstimulated cells from all the subgroups. Many of the individual values fell above the upper limit of the test range (Table 3). In antigen-specific responses, the levels of these cytokines dropped by between 0–200 up to 1000-fold in all the subgroups. Baseline TNFα medians were higher in both subgroups with egg positive stools (egg^+^IgE ^hi^ and egg^+^IgE^lo^). In HIV-p24 challenged cells, both groups with a high IgE profile (egg^+^IgE ^hi^ and egg^-^IgE^hi^ ) had higher TNFα medians (Table 4), while in Ascaris-worm challenged cells, TNFα median was highest in the egg^+^IgE^hi^ subgroup (Table 5). The group without evidence of helminth infection (egg^-^IgE^lo^) had the lowest or undetectable levels of all pro-inflammatory (IL-6, IL8 and TNFα) median values in response to HIV-p24 (Table 4) and *Ascaris*-stimulation (Table 5).

**Table 3:** **Cytokine levels in unstimulated PBMCs of HIV-infected subgroups**

Cytokine (pg/ml)	Phenotype subgroups			
	Median (minimum-maximum)			
	Egg^+^IgE^hi^(n = 10)	Egg^+^IgE^lo^(n = 10)	Egg^-^IgE^hi^(n = 10)	Eggs^-^IgE^lo^(n = 9)
**Pro-inflammatory**				
IL-6	10762 (9.2–12044)	9158 (4066–12985)	10253 (192–12547)	12671 (1267–>10000)
IL-8	8666 (605–10845)	8653 (5927–11872)	10723 (6785–11994)	11934 (7936–13130)
TNFα	153 (0.46–469)	200 (19–1012)	68 (23–2759)	81(16–398)
**Anti-inflammatory**				
IL-1RA	5369 (50–>10000)	6058 (2359–>10000)	>10000 (426–>10000)	>10000 (283–>10000)
**Type one (Th1)**				
IL-2	0 (0–0)	1.4 (0–6.8)	0 (0–5.8)	1.09 (0–5.9)
IFNγ	0 (0–1.1)	2.0 (0–252)	1.2 (0.12–808)	0.4 (0–3.5)
IP-10	41 (4–118)	174 (12–7739)	48 (5–1051)	113 (0–640)
GM-CSF	105 (0–177)	85 (15–476)	47 (18–1997)	54 (8–292)
**Type two (Th2)**				
IL-4	20 (3–21)	10 (3–23)	20 (10–48)	16 (13–18)
IL-5	0.1 (0–0.1)	0.08 (0–2.7)	0.2 (0.1–232)	(0.1–0.2)
IL-13	0 (0–11)	0.16 (0–161)	3.71 (0–2943)	0 (0–0)
**Regulatory**				
IL-10	31 (0–280)	116 (29–406)	40 (4–403)	147 (3–801)

Egg–Faecal helminth eggs, IgE–*Ascaris lumbricoides*-specific IgE, IgE hi/lo–high/low IgE in serum, IL-interleukin, IL–1RA interleukin-1 receptor antagonist; IFNγ–interferon gamma; IP-10–interferon-inducible protein 10, TNFα–Tumour necrosis factor alpha, GM-CSF–granulocyte-monocyte stimulation factor, RPMIc–omplete RPMI medium.

**Table 4:** **Cytokine production in response to HIV-1 p24 challenge**

Cytokine (pg/ml)	Phenotype subgroups			
	Median (minimum-maximum)			
	Egg^+^IgE^hi^(n = 10)	Egg^+^IgE^lo^(n = 10)	Egg^-^IgE^hi^(n = 10)	Egg^-^IgE^lo^(n = 9)
**Pro-inflammatory**				
IL-6	46.5 (0–1410)	866 (0–4599)	573 (0–12483)	0 (0–1234)
IL-8	864 (0–5293)	1032 (0–3388)	591 (0–3690)	0 (0–1076)
TNF-alpha	20 (0–250)	7 (0–53)	30 (0–1380)	2.9 (0–217)
**Anti-inflammatory**				
IL-1RA	5.1 (0–218)	22 (0–516)	0 (0–412)	11 (0–318)
**Type one (Th1)**				
IL-2	0 (0–0)	0.40 (0–2.1)	0 (0–3.1)	0 (0–4.6)
IFN-γ	0 (0–0)	11.1 (0–66)	1.9 (0–497)	1.4 (0–63.1)
IP-10	9.3 (0–104)	23.5 (0–101)	40.2 (0–1708)	78.9 (0–875)
GM-CSF	0 (0–96)	13.5 (0–65)	8.5 (0–227)	0 (0–96)
**Type two (Th2)**				
IL-4	0 (0–4.7)	0 (0–4.8)	0 (0–7)	0 (0–2.3)
IL-5	0 (0–0)	0 (0–1.1)	0 (0–1.8)	0 (0–0.1)
IL-13	0 (0–2.2)	2.3 (0–45)	0(0–11.4)	0 (0–2.2)
**Regulatory**				
IL-10	10.2 (0–110)	33.9 (0–95)	8.7 (0–305)	11.2 (0–105)

Egg–Faecal helminth eggs, IgE–*Ascaris lumbricoides*-specific IgE, IgE hi/lo- high/low IgE in serum; IL–interleukin, IL-1RA–interleukin-1 receptor antagonist; IFNγ interferon gamma; IP-10 interferon-inducible protein 10, TNFαTumour– necrosis factor alpha, GM-CSF-granulocyte-monocyte stimulation factor, RPMIc–omplete RPMI medium.

**Table 5:** **Cytokine production responses to** ***Ascaris*** **crude antigen stimulation in HIV-infected subgroups**

Cytokine (pg/ml)	Phenotype subgroups			
	Median (minimum-maximum)			
	Egg^+^IgE^hi^(n = 10)	Egg^+^IgE^lo^(n = 10)	Egg^-^IgE^hi^(n = 10)	Eggs^-^IgE^lo^(n = 9)
**Pro-inflammatory**				
IL-6	638 (0–1757)	2087 (0–6514)	632 (0–12878)	318 (0–1919)
IL-8	231 (0–4603)	1250 (0–4112)	31 (0–3620)	0 (0–960)
TNF-alpha	123 (0–219)	61 (0–219)	66 (11–1511)	29 (0–389)
**Anti-inflammatory**				
IL-1RA	44 (0–6564)	823 (224–988)	61 (19–761)	101 (123–999)
**Type one (Th**_**1**_ **)**				
IL-2	0.6 (0–4.4)	1.7 (0–5.5)	2.8 (0–8.2)	0.4 (0–165)
IFN-gamma	0 (0–13.8)	1.8 (0–99)	14 (0–600)	0.2 (0–110)
IP-10	0(0–87)	0 (0–4.6)	0 (0–154)	0 (0–119)
GM-CSF	16 (0–218)	33 (0–517)	38 (0–452)	11 (326)
**Type two (Th**_**2**_ **)**				
IL-4	0 (0–8.4)	4.4 (0–12.2)	6.6 (0–103)	2.1 (0–26)
IL-5	0.2 (0–3.8)	0.2 (0–23)	4.7 (0–460)	0 (0–96)
IL-13	5.5 (0–28)	18 (2.5–355)	81 (0–2120)	2.4 (0–1903)
**Regulatory**				
IL-10	61 (0–138)	77 (0–303)	52 (0–324)	33 (0–248)

Egg–Faecal helminth eggs, IgE *Ascaris lumbricoides*-specific IgE, IgE hi/lo–high/low IgE in serum; IL–interleukin, IL-1RA–interleukin-1 receptor antagonist; IFNγ–interferon gamma; IP-10–interferon-inducible protein 10, TNFα–Tumour necrosis factor alpha, GM-CSF granulocyte–monocyte stimulation factor, RPMIc–omplete RPMI medium.

### Anti-inflammatory cytokines

The baseline anti-inflammatory cytokine- IL-1RA levels were also very high. Both subgroups with positive stool eggs (egg^+^IgE^hi^ and egg^+^IgE^lo^) had relatively lower median levels of this cytokine compared to the two without stool eggs. For the latter subgroups, the median values fell outside the upper limit (>10 000 pg/ml) (Table 3). In HIV-p24 and *Ascaris* stimulated assays, IL-1RA was lower in both subgroups with a high IgE profile (Tables 4 and 5). The egg^+^IgE^lo^ subgroup had a significantly higher level (p = 0.03) of IL-1RA in response to HIV-1 p24 challenge.

### Type 1 cytokines

Generally, the baseline levels of IL-2 and IFNγ were low (<2 pg/ml) while those of IP-10 and GMSF were higher (>10 pg/ml) in all assays. All individuals in the egg^+^IgE^hi^ subgroup had undetectable baseline levels of the lymphoproliferative cytokine, IL-2 and a trend for lower responses between this subgroup and the egg^-^IgE^lo^ group (p = 0.07) was observed. Similarly, in the egg^+^IgE^hi^ subgroup the antiviral cytokine, interferon gamma (IFNγ) was undetected in all except only one participant with 1,14 pg/ml IFNγ. Median IFNγ production by unstimulated cells differed significantly between this subgroup and the egg^+^IgE^lo^ (p = 0.02) and the egg^-^IgE^hi^ (p = 0.04) subgroups and there was a trend for a decrease in this subgroup when compared to the egg^-^IgE^lo^ subgroup (p = 0.09) (Table 3). Overall, all type 1 cytokines responses to HIV-p24 were lowest in the cells of individuals in the egg^+^IgE^hi^ subgroup (except GMCSF, which was also low in the egg^-^IgE^lo^ subgroup). In HIV-1p24 and *Ascaris* antigen stimulated assays, levels of IFNγ were undetectable in this egg^+^IgE^hi^ subgroup (Tables 4 and 5). Baseline IP-10 levels were lower in both subgroups with high IgE. Individuals in the egg^+^IgE^lo^ subgroup had the highest levels of all baseline type 1 cytokines (except for GMCSF) (Table 3), while they produced significant levels (p = 0.05) of IFNγ in response to HIV-1 p24 challenge (Table 3).

### Type 2 and regulatory cytokines

IL-5 and IL-13 cytokine levels were very low (less than 1 pg/ml) or not detectable in unstimulated and HIV-1 p24 stimulated cell assays and in the latter all type 2 cytokines were undetectable except in the egg^+^IgE^lo^ group with an IL-13 median of 2.25 pg/ml. However, IL-5 and IL-13 cytokine responses to *Ascaris* worm challenge were detectable in all subgroups with evidence of helminth infection (eggs or high IgE). Baseline IL-4 levels were higher in both groups with a high IgE profile and lowest in the egg^+^IgE^lo^ subgroup. In HIV-p24 stimulated cells, all type 2 cytokines were low or undetectable, while in *Ascaris* stimulated cells, IL-4 was undetectable in the egg^+^IgE^hi^ subgroup. Higher baseline (Table 3) and HIV-p24 (Table 4) levels of the regulatory cytokine-IL-10 were associated with low IgE subgroups. The egg^+^IgE^lo^ subgroup had the lowest cytokine-IL-10.

## Discussion

Chronic helminth infections have been associated with suppressed T cell proliferative responses against parasite antigens as well as other unrelated antigens [20]. Likewise it has been shown that the proliferative capacity of cells from individuals with HIV or both HIV and helminth infections is attenuated [1, 4]. It has also been suggested that helminth infections result in increased proinflammatory cytokines while type 2 cytokines are a classic feature of these infections [1, 3]. Predominance of type two cytokines results in downregulation of the type 1 response, which is crucial for the control of viral infections including HIV. The present study aimed to determine if peripheral blood mononuclear cells from HIV-infected and negative South African adults with dual (HIV and helminths) infection have decreased ability to proliferate in response to antigens and mitogens using Ki67 expression as a marker, and whether cytokine responses are altered in the HIV and helminth co-infected individuals.

The results showed that individuals with an egg^+^IgE^hi^ phenotype had a tendency to increased immune activation, viraemia, impaired antigen specific responses and retained potent proliferative responses to mitogen. The percentage expression of Ki67 in response to HIV-1 p24, Streptokinase and *Ascaris* worm antigens were lower than those of PHA. In this subgroup there were increased background levels of Ki67 and CTLA-4 in unstimulated cell cultures in the majority of individuals. Simultaneous increase of both Ki67 and CTLA-4 indicates activated cells arrested at the G1 phase and not necessarily proliferating cells [18]. In this study, the expression of Ki67 was significantly higher among the HIV-infected compared to the HIV-uninfected individuals, which is in keeping with increased cell turnover during chronic infections [9]. Furthermore, both Ki67 and CTLA-4 expression by individuals in the HIV^+^ egg^+^IgE^hi^ subgroup were increased in the absence of stimulating antigen. This strongly suggests immune activation and corroborates our previous findings wherein all activation markers were highly increased in this subgroup [14]. Among the HIV-uninfected, HIV-egg + IgE hi subgroup had the lowest median CD4. However, as can be seen in Table 3 one of the patients included in this group had CD4 count of 0,13 cells/ml. Since the group only comprises of altogether five individuals this could have a significant impact on the results. Alternatively, it should be noted that helminth infections are also documented to decrease the CD4+ cell population [21].

The findings of lower proliferative responses to specific antigens and strong mitogen responses in the dually infected HIV + egg^+^IgE^hi^ individuals agree with previous reports that HIV and helminths selectively target and downregulate specific responses directed at them as well as other cognate antigens [1, 17]. In addition, HIV tat was shown to block CD26 (a costimulatory molecule) thereby attenuating effective transduction of the stimulation signals [22]. Likewise, both HIV and helminths are documented to downregulate expression of another costimulatory molecule, CD28 [1], thus inducing generalised and specific anergy to themselves and other infecting pathogens.

All HIV-1 positive subgroups with some evidence of helminth exposure (egg^+^IgE^hi^, egg^+^IgE^lo^ and egg^-^IgE^hi^ subgroups) had higher Ki67 responses to mitogen challenge when compared to the egg^-^IgE^lo^ group (without helminth infection), implying that their cells were viable and retained their capacity to proliferate in response to non-specific stimulation. However, individuals with the egg^+^IgE^hi^ phenotype showed decreased proliferative (Ki67) responses to HIV-1 p24, streptokinase and *Ascaris* worm antigens although these differences were not statistically significant. This may suggest that antigen specific responses are compromised in the presence of HIV- helminth co-infections [1]. We found that this phenomenon is prominent in individuals who excrete eggs and respond with a high specific IgE phenotype. While the Ki67 responses to PHA were high in all helminth-exposed subgroups, egg excretion (egg^+^IgE^hi^ and egg^+^IgE^lo^) was also accompanied by increased PHA-induced CLTA responses in the HIV-infected group. In the HIV-uninfected group, both Ki67 and CTLA-4 were increased in response to the recall antigen in both egg positive subgroups. This may suggest that the presence of eggs (indicating current and ongoing infection) is associated with increased non-specific and recall activation and inherent regulation.

The results also showed that baseline pro-inflammatory cytokines IL-6 and IL-8 were increased in all the subgroups and there was a marked decrease of these markers in antigen-stimulated assays. High TNFα levels were associated with egg excretion or with high *Ascaris*-specific IgE in response to antigen-stimulation. Egg excretion may be associated with ongoing inflammatory processes [23] and increased TNFα levels were found in the stool egg excretors in this study. Particularly, the egg^+^IgE^hi^ subgroup showed higher levels of TNF in unstimulated, HIV-p24 and *Ascaris* antigen stimulated cells. TNFα alone facilitates HIV transcription via the activation of the nuclear factor kappa-beta while IL-6 and TNFα act synergistically to promote HIV replication [3]. In this study, while IL-6 and IL-8 were very high in all the subgroups at baseline, increased TNFα levels were associated with helminth infection (either egg positivity at baseline or with high *Ascaris*-specific IgE profile in antigen-stimulated assays). This observation is in support of the proposition that dual infection with HIV and helminths provides a favourable milieu for HIV propagation [1, 3]. This assertion is supported by our finding that all pro-inflammatory cytokines were lowest in the subgroup with single HIV-1 infection (egg^-^IgE^lo^) in response to HIV-1 and *Ascaris* worm challenge.

Concurrently, lower anti-inflammatory cytokine (IL-1RA) levels were observed in egg excreting subgroups. In HIV-p24 and *Ascaris* antigen challenged cells, IL-1RA was lower in high IgE subgroups. These findings suggest that individuals infected or exposed to helminths have lowered anti-inflammatory responses. The singly HIV-infected subgroup (egg^-^IgE^lo^) had the lowest or undetectable levels of all the three proinflammatory cytokines, demonstrating that dual infection leads to both enhanced pro-inflammatory and impaired anti-inflammatory reactions, particularly in the egg^+^IgE^hi^ phenotype, supporting a role of helminths in promoting HIV replication [1, 3] .

The type 1 cytokines IFNg, IL-2 were generally low in this study. Nonetheless, lower type 1 cytokines, more importantly IFNγ and IL-2 in the egg^+^IgE^hi^ subgroup were detected, and can be a contributor to enhanced HIV disease whose control is largely mediated by these cytokines. IL-2 is a major lymphoproliferative cytokine that also drives differentiation of lymphocytes into armed effector cells while IFNγ, as antiviral cytokine, plays a critical role in killing of virally-infected cells [24]. Production of these two cytokines was lower, not only in unstimulated PBMCs but also in cells stimulated with the HIV-1 p24 antigen. Lower levels of the lymphoproliferative cytokine- IL-2 in the egg^+^IgE ^hi^ subgroup are also in keeping with low antigen-specific proliferative capacity in these individuals as discussed above.

Furthermore, the significant reduction of IL-2 and IFNγ with a trend towards decreased antigen-specific proliferative responses in the egg^+^IgE^hi^ subgroup corresponds with the notion that defective IL-2 production is associated with advancing HIV immunodeficiency [6]. However, the changes in expression of type1/type2 cytokines during HIV –helminth co-infection has been challenged and inconsistent findings reported recently [25]. In addition, the simultaneous reduction of IL-2 and IFNγ in this group concurs with the findings by Sousa *et al.* [26] that HIV-1 progression is associated with reduction of the population of CD4^+^ lymphocytes with potent proliferative capacity and ability to produce IL-2 and IFNγ. In the same context, the results of the present study corroborate the findings of a tendency towards reduction of CD4^+^ lymphocyte numbers in the egg^+^IgE^hi^ subgroup in our previous paper [14]. In addition, our results showed that CTLA-4 expression was increased in the egg^+^IgE^hi^ subgroup. CTLA-4 inhibits production of IL-2 among other down-modulatory functions [24]. These provide an immunological basis that is in line with a negative interaction between HIV and helminth co-infection [1, 3] particularly in the egg^+^IgE^hi^ individuals.

While IL-4 responses to antigen stimulation were spurious, at baseline this type 2 cytokine was higher in high IgE responder subgroups. High IgE and IL-4 are classic type 2 anti-helminth responses. IL-10 levels were lower in these subgroups. These high IgE responder groups displayed lower type 1 and anti-inflammatory cytokines as discussed above. This lends further support to the hypothesis that helminth infection induces a type 2 biased cytokine response that dampens type 1 cytokine production and impairs anti-HIV immune response [1, 3, 27].

Taken together these findings suggest that egg excretion and/or high *Ascaris* specific IgE associated with lower anti-inflammatory, higher pro-inflammatory and IL-4 plus lower type 1 cytokines. This cytokine profile, which was particularly prominent in the egg^+^IgE^hi^ subgroup, accompanied by the reported tendency to impaired specific and recall antigen responses could be deleterious to immune responses to HIV.

The egg^+^IgE^lo^ subgroup on the other hand showed a favourable profile, characterized by higher IFNγ, IL-2, lower IL-4 and higher IL-10. The egg^+^IgE^lo^ subgroup had the highest levels of type 1 cytokines (except GMCSF which was highest in the egg^+^IgE^hi^ individuals); the lowest IL-4 at baseline and particularly produced a strong IFNγ response and significant anti-inflammatory response to HIV-p24 challenge. This cytokine profile concurs with previous findings that these individuals mimic the modified Th2 phenotype [14, 28]. This suggests that some individuals who respond with a low IgE to helminth infections may be better able to control HIV infection, while those with a high IgE and excrete eggs are more likely to respond poorly to HIV infection.

The present study had some limitations. The costs of the laboratory tests did not allow for testing of all available samples. In addition the unavailability of ART limited the recruitment of potential HIV-uninfected participants, hence, the small numbers in some subgroups. As a result some of the results could not be validated through statistical analysis. For ethical reasons, limited amount of blood could be drawn from the HIV-infected participants because a proportion of them would be immunocompromised already. Consequently, cells had to be split up for assessment of surface activation markers and the remainder used for setting up cultures for proliferation and subsequent supernatant cytokine assays. Furthermore, the study used human AB male serum for cytokine analyses and not serum-free medium. Nevertheless, human AB male serum has been shown to preserve the proliferation capacity of cells [29], which was one of the aims of this paper. In addition, serum supplemented media has been widely used in recommended protocols [30]. Another limitation of this study was difficulty to find balance for optimum incubation period for all the cytokines analysed, against the requirement for longer incubation to expand antigen-specific cellular responses [31]. Only two cytokines were investigated (IFNγ and IL-4) from which an optimum of five days was found for antigen-specific responses. This culture period may also have increased activation-induced apoptosis, particularly in cells from HIV-infected persons that are documented to be highly susceptible to programmed cell death [32]. The sharp drop in proinflammatory cytokines, in particular could be attributable to this limitation.

## Conclusion

Notwithstanding the limitations in this work, the findings suggest that dual HIV/helminth infection with egg excretion and/or high *Ascaris* IgE phenotype may be linked with poor proliferative capacity and deleterious cytokine profile with regards to HIV control. This study suggests that dual infection with helminths and HIV may induce impaired antigen-specific responses and unfavourable cytokine profiles in individuals with a high parasite-specific IgE and egg excretion phenotype. Individuals who responded with low IgE to helminths are likely to retain competent immune profile in terms of HIV responses. It is therefore important that workers who investigate the intricate relationship between HIV and helminths are cognizant of other host factors such as the different helminth-infection response phenotypes described in our work and by others [17].

### Finally, this study was not designed to look at associations but rather to explore

differences in the different subgroups given the expression of various cellular markers from an extensive laboratory analysis. Secondary analysis of this can in future be used to explore possible associations and control for multiple comparisons for bigger sample sizes as the latter was one of the study limitations. However, this work adds new knowledge, particularly in the South African context, to the still controversial role of helminth co-infection on HIV disease.

## Authors’ original submitted files for images

Below are the links to the authors’ original submitted files for images. Authors’ original file for figure 1 Authors’ original file for figure 2
