# Supplementary figures and images for: Proliferative capacity and cytokine production by cells of HIV-infected and uninfected adults with different helminth infection phenotypes in South Africa
Source: BMC Infect Dis. 2014 Sep 11;14:499. doi: 10.1186/1471-2334-14-499 (PMC4262143; doi:10.1186/1471-2334-14-499)

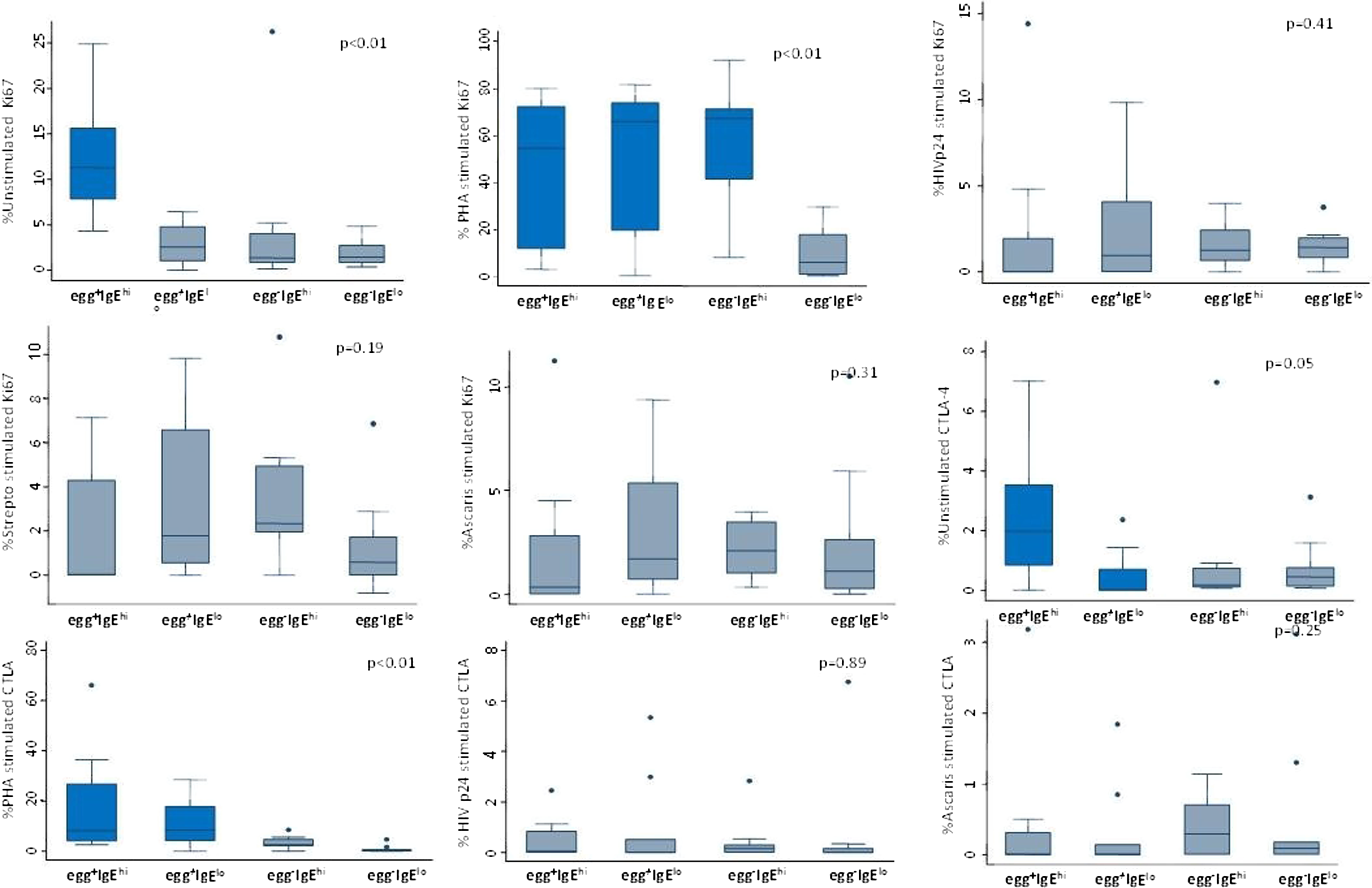

Supplement: Supplementary file 1 — Authors’ original file for figure 1 [file 12879_2013_4052_MOESM1_ESM.tif]

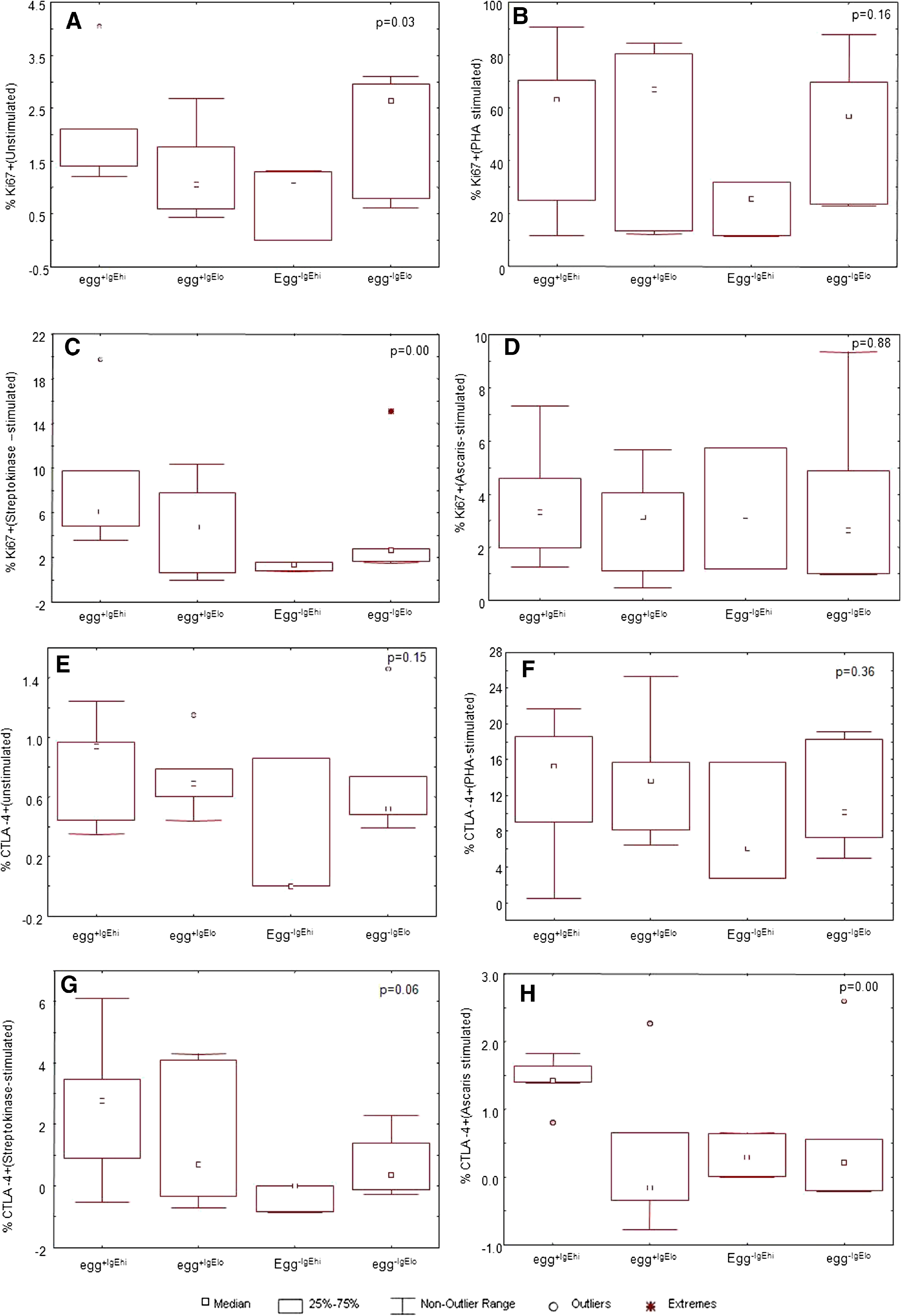

Supplement: Supplementary file 2 — Authors’ original file for figure 2 [file 12879_2013_4052_MOESM2_ESM.tif]
